# Streamlining antibiotic use in community acquired pneumonia: A quality improvement initiative

**DOI:** 10.1002/jhm.70283

**Published:** 2026-02-10

**Authors:** Claire E. Ciarkowski, Karen Howard, Hannah Imlay, Chaorong Wu, Christy L. Hopkins, Valerie M. Vaughn, Emily S. Spivak

**Affiliations:** ^1^ Department of Internal Medicine, Division of General Internal Medicine University of Utah Salt Lake City Utah USA; ^2^ Department of Pharmacy University of Utah Health Salt Lake City Utah USA; ^3^ Department of Internal Medicine, Division of Infectious Diseases University of Utah Salt Lake City Utah USA; ^4^ Veteran's Affairs Salt Lake City Health Care System Salt Lake City UT USA; ^5^ Department of Internal Medicine, Division of Epidemiology University of Utah Salt Lake City Utah USA; ^6^ Department of Emergency Medicine University of Utah School of Medicine Salt Lake City Utah USA

## Abstract

**Background:**

Evidence suggests a 3‐day total duration and early transition to oral therapy is safe in hospitalized patients with community acquired pneumonia (CAP)—though such care is not standard in the United States. To implement these evidence‐based practices, a multidisciplinary group led by a hospitalist and antimicrobial stewardship updated an order set targeted at non‐intensive care unit (ICU) patients with CAP.

**Objective:**

Here, we assess the impact of our intervention on diagnostic and antibiotic treatment measures and associated clinical outcomes.

**Methods:**

This is a retrospective, pre/post, observational quality improvement study of non‐ICU inpatients with CAP admitted to a single academic medical center. The order set defaulted to 1 day of ceftriaxone without empiric atypical coverage for most patients, automatic de‐escalation to oral amoxicillin on Day 2, a shortened total antibiotic duration of 3 days, and appropriate laboratory utilization. Primary outcomes were antibiotic duration and azithromycin use. Clinical outcomes were also assessed.

**Results:**

The pre (1/2019–12/2021, *n* = 777) and post (1/2022–12/2023, *n* = 1416) intervention periods were compared using generalized linear models and interrupted time series adjusted for known confounders. After implementation of the orderset, total antibiotic duration (6–5 days, adjusted rate ratio: 0.92, 95% confidence interval [CI]: 0.89–0.95, *p* < 0.01) and azithromycin use (62.4% [485/777] to 39.4% [558/1416], adjusted odds ratio: 0.39, 95% CI: 0.33–0.47, *p* < 0.01) were significantly lower. Clinical outcomes were not significantly different postintervention.

**Conclusions:**

An order set backed by stewardship and hospitalists improves appropriate antibiotic use, showing that ongoing data‐driven refinements can sustain stewardship and support the safety of shorter, narrower antibiotics for CAP in real‐world care.

## Visual Abstract

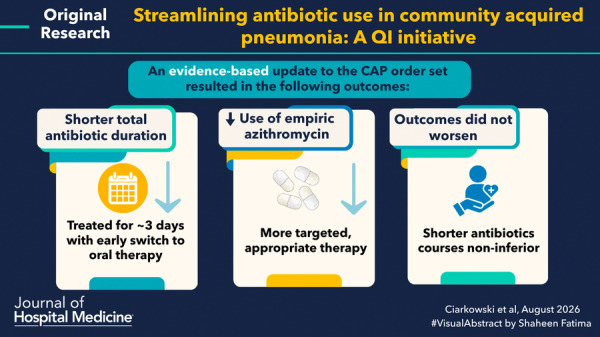

## INTRODUCTION

Community‐acquired pneumonia (CAP) remains a leading cause of hospitalization and economic burden with over 1.5 million adults hospitalized annually in the United States.^1^, ^2^, ^3^ Inappropriate diagnosis of CAP is suspected to occur in at least one in eight patients due to subjective symptoms and radiologic findings that could represent multiple etiologies.^4^, ^5^ Overdiagnosis of CAP and treatment for long durations lead to increased rates of antibiotic‐associated adverse events.^6^, ^7^

The Infectious Diseases Society of America (IDSA) recommends developing and implementing locally adapted guidelines to improve process of care variables and relevant clinical outcomes.^2^ University of Utah Health first implemented a pneumonia order set for patients hospitalized with CAP in 2017, which led to cost savings and decreased duration of intravenous (IV) and total antibiotics with similar clinical outcomes.^7^, ^8^ Since then, additional literature has emerged supporting 3 days of antibiotics in nonintensive care unit (ICU) patients with CAP.^9^, ^10^ Literature questioning the necessity of macrolides for all patients has sparked clinical debate. Although atypical coverage is still recommended in IDSA and American Thoracic Society (ATS) CAP guidelines for all hospitalized patients, existing data suggest that a beta‐lactam and macrolide may not be needed for non‐ICU CAP patients, as there is no survival benefit in this population.^11^, ^12^, ^13^ The benefit of adding “atypical coverage” with azithromycin is only seen in patients with confirmed infection due to atypical organisms, which are uncommon, and in patients with more severe disease.^14^, ^15^ Some guidelines continue to recommend beta‐lactam monotherapy for hospitalized ward patients with severe CAP (CURB‐65: 3‐5; PSI: 5).^16^, ^17^ Based on review of the literature and local epidemiology, the CAP order set at University of Utah Health was updated in late 2021 to focus on narrowing empiric antibiotics, shortening total duration of therapy and promoting appropriate laboratory utilization for non‐ICU patients with CAP. We aimed to evaluate the impact of our CAP order set on antibiotic use, laboratory utilization, and clinical outcomes for patients admitted with CAP. We hypothesized that our updated guidance and order set would lead to decreased total antibiotic duration and narrowed antibiotic use without adversely affecting clinical outcomes.

## METHODS

### Setting and design

University of Utah Health is a 678 (333 medicine‐surgical) bed academic medical center located in Salt Lake City, Utah with over 9000 admissions to the hospitalist service annually. We conducted a retrospective, observational pre–post quality improvement study of inpatients admitted with CAP to non‐ICU units. This study examined the impact of a quality improvement intervention and was deemed exempt from the University of Utah Institutional Review Board. We followed standards for quality improvement reporting excellence reporting guidelines for quality improvement studies (Supporting Information S1: Appendix).^18^

### Participants

Our study included hospitalized adult patients with a discharge International Classification of Diseases, Tenth Revision, Clinical Modification (ICD‐10‐CM) code of pneumonia (see Supporting Information S1: Appendix for Epic® CAP Grouper inclusion) who also received antibiotics within 48 h of admission. Patients were excluded if they were less than 18 years old at the time of discharge, admitted to an ICU within 24 h of admission, had a lifetime history of cystic fibrosis or a solid organ or bone marrow transplant, or had an encounter diagnosis of coronavirus disease 2019 (COVID‐19) (see Supporting Information S1: Appendix for all relevant exclusion ICD‐10‐CM codes). Patients were also excluded if they received more than 14 days of antibiotics or 2 or fewer days of antibiotics. We excluded patients admitted to our affiliated cancer hospital.

### Intervention

A multidisciplinary group composed of members from hospital medicine, intensive care/pulmonology, emergency medicine, infectious disease/antimicrobial stewardship, pharmacy and information technology support initially launched a clinical decision support system‐triggered CAP order set in 2017.^8^ This initial order set for non‐ICU patients had an automatic switch from a single dose of IV ceftriaxone and azithromycin to oral cefuroxime alone for a total antibiotic duration of 5 days.^8^ In 2021, further discussions led by hospital medicine and antimicrobial stewardship were held to update the existing order set for non‐ICU patients to encourage narrowing empiric antibiotics without empiric atypical coverage for most patients and a shortened duration of therapy. The updated order set included the following changes:

- Empiric IV ceftriaxone with an automatic switch to oral amoxicillin for a total duration of 3 days.
- Remove the order for azithromycin for most patients.
- Levofloxacin for 3 days for patients with a severe beta‐lactam allergy.
- Remove order for blood cultures.
- De‐select the standing order for *Streptococcus pneumoniae* urine antigen.
- Continue standing order for procalcitonin.
- Continue standing order for *Legionella* urine antigen.

Please see the Supporting Information S1: Appendix for a complete table of changes and associated outcomes. Most patients with uncomplicated CAP are admitted to the hospitalist service at University of Utah Health, so efforts on education and feedback regarding proposed changes to the electronic medical record focused on this group as well as the emergency department (ED). Specifically, education co‐led by the antimicrobial stewardship program with open discussion was held during a weekly hospitalist meeting and education was sent in email updates. Medicine pharmacists were also educated on order set changes and worked with antimicrobial stewardship pharmacists to remind hospitalists on rounds if their patient qualified for a shorter antibiotic duration. Updates to the order set went live on November 11, 2021.

### Measures and analysis

The preintervention period was defined as patient encounters from 1/2019 to 12/2021 while the postintervention period included patient encounters from 1/2022 to 12/2023. The postimplementation period started 1 month after go‐live to ensure all education and roll out of the intervention was complete. Demographic and clinical characteristics were compared between the pre‐ and postintervention period using the Mann–Whitney *U* test for continuous variables and the *χ* ^2^ test for categorical variables.

Primary outcomes included total antibiotic duration and percentage of encounters with azithromycin use in the pre‐versus postperiod. Secondary outcomes included the percentage of patients receiving fewer than 5 days of antibiotics, the percentage prescribed amoxicillin, 30‐day ED visits, 30‐day readmissions, 30‐day mortality, combined 30‐day readmission and 30‐day mortality, LOS, and ICU transfers. To assess the impact of our intervention, we used generalized linear models adjusted for age, Charlson Comorbidity Index (CCI), and severity index (defined as presence of ≥2: respiratory rate >20, heart rate >90, blood pressure <90 mmHg, temperature >38°C or <35°C). Severity index without white blood count was used instead of systemic inflammatory response syndrome due to data architecture changes in the electronic medical record that made data capture for this variable inaccurate. Gamma distribution with a log link function was used for total antibiotic duration, and LOS and logistic regression was used for other variables. For the primary outcomes, an interrupted time series analysis was conducted to compare the pre‐ and postintervention periods, accounting for the effect of time (month) and the interaction between the intervention and time. The analysis also controlled for age, CCI, and severity index.

Finally, we evaluated predictors of 30‐day readmission and 30‐day mortality across the entire period using a post hoc multivariable regression analysis including azithromycin use, amoxicillin use, antibiotic duration, age, sex, severity index, CCI, history of heart failure, history of chronic obstructive pulmonary disease, and respiratory virus season defined as months including November through February. All analyses were performed using R Statistical Software (v4.4.0; R Core Team 2024).

## RESULTS

A total of 777 patients were included in the preintervention period. Due to significant hospital growth (addition of approximately 120 acute medicine beds around 2021) and impacts from the COVID‐10 pandemic on CAP admissions in the preintervention period, the postintervention period included 639 additional encounters for a total of 1416 postintervention encounters. Patient characteristics are summarized in Table 1. The CAP order set was used in approximately 38% of visits in both periods (Table 1). Patient demographics were similar in both periods with more patients having chronic obstructive pulmonary disease (COPD) in the postperiod (Table 1).

**Table 1:** Patient characteristics.

Variable	Pre	Post	*p* value
Total (*n*)	777 (35.4)	1416 (64.6)	
Age, years; median (IQR)	64.0 (49.0–77.0)	64.0 (50.0–76.0)	1.0
Sex; *n* (%)			0.94
Male	433 (55.7)	793 (56.0)	
Female	344 (44.3)	623 (44.0)	
Race; *n* (%)			0.18
White	605 (78.0)	1048 (74.0)	
Other	148 (19.1)	327 (23.1)	
Black	17 (2.2)	32 (2.3)	
Asian	6 (0.8)	9 (0.6)	
Ethnicity; *n* (%)			0.47
Not Hispanic/Latino	664 (85.5)	1225 (86.5)	
Hispanic/Latino	78 (10.0)	142 (10.0)	
Unknown/information not available	35 (4.5)	49 (3.5)	
Comorbidities present, *n* (%)			
COPD	31 (4.0)	156 (11.0)	< 0.01
Heart failure	48 (6.2)	113 (8.0)	0.14
Dementia	4 (0.5)	19 (1.3)	0.11
Severity index,^a^ *n* (%)	538 (69.2)	949 (67.0)	0.31
CCI score, median (IQR)	5.0 (3.0 to 8.0)	5.0 (2.0 to 7.0)	0.16
CAP order set use, *n* (%)	293 (37.7)	547 (38.6)	0.71
Laboratory, *n* (%)			
Legionella, total ordered	562 (72.3)	952 (67.2)	0.04
Legionella, positive	4/562 (0.7)	5/952 (0.5)	0.73^b^
* Streptococcus pneumoniae* urine antigen ordered	563 (72.5)	646 (45.6)	< 0.01
Blood cultures ordered	489 (62.9)	765 (54.0)	< 0.01
Procalcitonin ordered	541 (69.6)	1007 (71.1)	0.50
Respiratory culture ordered	231 (29.7)	437 (30.9)	0.62

Abbreviations: CAP, community‐acquired pneumonia; CCI, Charlson Comorbidity Index; COPD, chronic obstructive pulmonary disease; IQR, interquartile range.

^a^ Defined by ≥ 2 of the following on Day 1 of admission: systolic blood pressure ≤ 90 mmHg, respiratory rate ≥ 20 breaths per minute, heart rate ≥ 90 beats per minute, or temperature > 100.4 °F (38°C) or < 96.8 °F (36°C).

^b^ Fisher's exact test.

**Figure 1:**
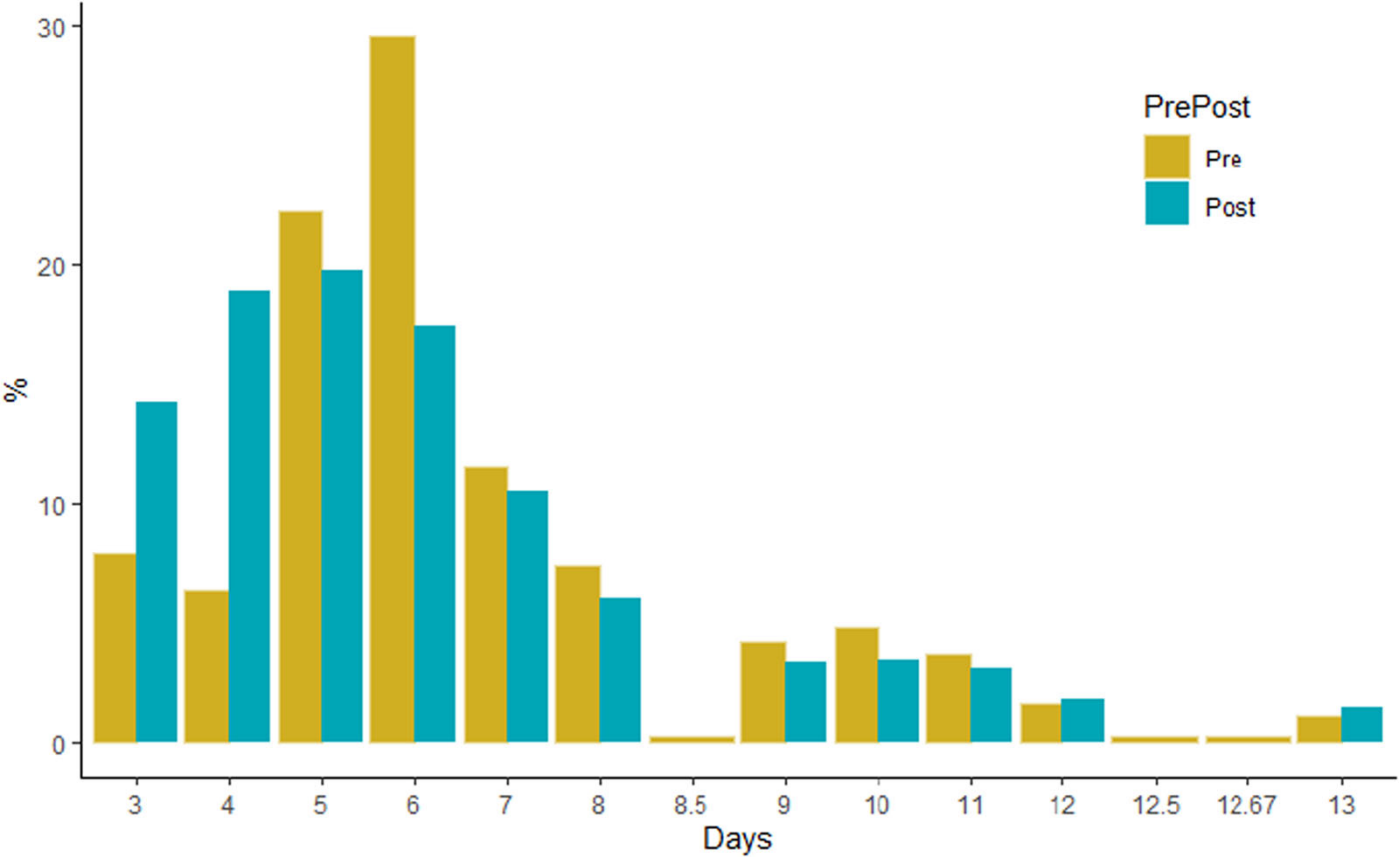
Distribution of antibiotic duration by percent. *Significantly more patients received fewer than 5 days of antibiotics in the postintervention period.

**Figure 2:**
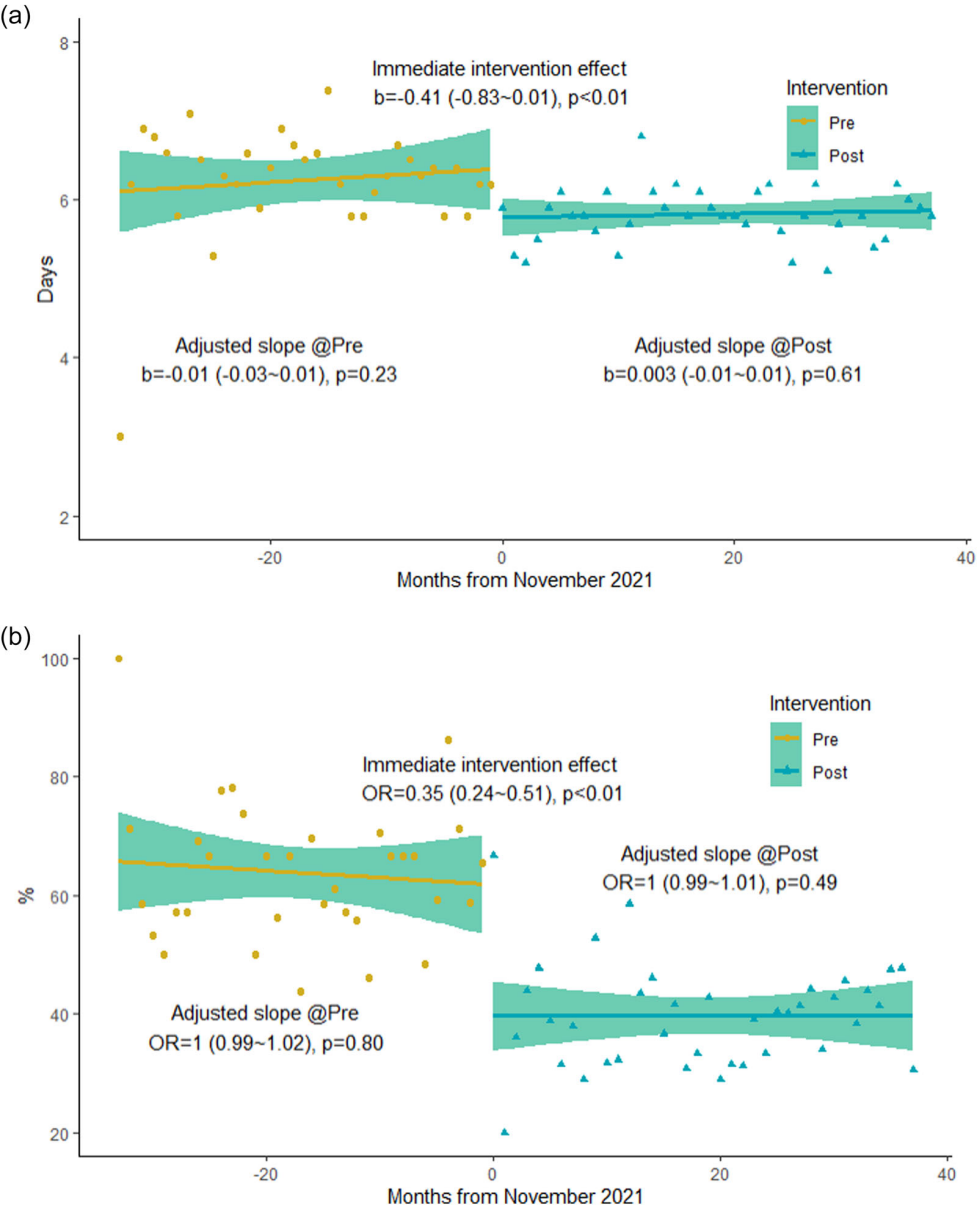
Interrupted time series analysis for primary outcomes. (a) Median days of total antibiotics per month. (b) Percent of patients who received Azithromycin at each month. Unadjusted smooth lines and the interrupted time series results for intervention and slope at pre and postinterventions. Each point represents the median total antibiotic duration per month. The line presents the unadjusted slope per intervention period. Each point represents the percentage of patients with an order for Azithromycin per month. The line presents the unadjusted slope per intervention period.

Median total antibiotic duration decreased from 6 days (interquartile range [IQR]: 5–7) in the preintervention period to 5 days (IQR: 4–7) in the postintervention period. After adjustment, antibiotic duration decreased by 8% from pre‐ to postintervention (adjusted rate ratio: 0.92, 95% confidence interval [CI]: 0.89–0.95, *p* <0.01) (Table 2). The total percentage of patients receiving 5 or fewer days of antibiotics increased from 14.2% (110/777) to 33.1% (469/1416). Adjusted odds of patients receiving 5 or fewer days of antibiotics were 2.98 times higher in the postintervention compared with those in the preintervention (95% CI: 2.37–3.75, *p* <0.01). A similar trend is observed in Figure 1. The percentage of patients who received azithromycin decreased from 62.4% (485/777) to 39.4% (558/1416) (adjusted odds ratio [aOR]: 0.39, 95% CI: 0.33–0.47, *p* <0.01) (Figure 2). Amoxicillin use increased from 5.1% (40/777) to 51.1% (723/1416). After adjustment, the odds of Amoxicillin use were 19.34 times higher in the postintervention compared with that in the preintervention (95% CI: 13.83–27.05, *p* <0.01). Interrupted time series analysis showed an immediate intervention effect for both primary outcomes (Figure 2).

**Table 2:** Descriptive statistics comparing pre‐ and postintervention periods and adjusted rate ratios or odds ratios for primary and secondary outcomes.

Antibiotic use	Pre	Post	aOR/aRR^a^	95% CI	*p* value
*Primary outcomes*	*N* = 777	*N* = 1416			
Total duration, days; median (IQR)	6.0 (5.0–7.0)	5.0 (4.0– 7.0)	0.92	0.89–0.95	< 0.01
Azithromycin ordered; *n* (%)	485 (62.4)	558 (39.4)	0.39	0.33–0.47	< 0.01
*Secondary outcomes*					
Antibiotic duration					
≤5 days; *n* (%)	110 (14.2)	469 (33.1)	2.98	2.37–3.75	< 0.01
≤4 days; *n* (%)	61 (7.9)	201 (14.2)	1.92	1.42–2.6	< 0.01
Amoxicillin ordered; n (%)	40 (5.1)	723 (51.1)	19.34	13.83–27.05	< 0.01
LOS, days; median (IQR)	4.0 (3.0–7.0)	4.0 (3.0–7.0)	1.03	0.96–1.11	0.39
ICU transfer during admission; *n* (%)	49 (6.3)	50 (3.5)	0.65	0.44–0.96	0.01
Combined 30‐day readmission and mortality	100 (12.9)	195 (13.8)	1.12	0.86–1.45	0.40
30‐day ED visit	97 (12.5)	233 (16.5)	1.42	1.1–1.84	0.01
30‐day readmission	49 (6.3)	133 (9.4)	1.59	1.13–2.24	0.01
30‐day mortality	54 (6.9)	69 (4.9)	0.71	0.49–1.03	0.07

Abbreviations: aOR, adjusted odds ratio; aRR, adjusted rate ratio; CI, confidence interval; CCI, comorbidity index; ED, Emergency Department; ICU, intensive care unit; IQR, interquartile range; LOS, length of stay.

a Rate ratio (antibiotic duration, length of stay) or odds ratio (all other variables), as appropriate, adjusted for age, CCI score, and Severity index.

Laboratory data are summarized in Table 1. The postintervention period was notable for statistically significant reductions in blood culture and *Streptococcus pneumoniae* urine antigen ordering. The percentage of patients with a procalcitonin order was similar in both time periods. Legionella urine antigen testing decreased from 72.3% (562/777) to 67.2% ([952/1416], *p* =0.04); however, total positive cases remained low (Table 1).

Thirty‐day emergency department visits and 30‐day readmissions increased in the postintervention period (12.5% [97/777] to 16.5% [233/1416], aOR: 1.42, 95% CI: 1.1–1.84, *p* = 0.02 and 6.3% [49/777] to 9.4 [133/1416], aOR: 1.59, 95% CI: 1.13–2.24, *p* = 0.01). Thirty‐day mortality was nonsignificantly lower (6.9% [54/777] to 4.9% [69/1416], aOR: 0.71, 95% CI: 0.49–1.03, *p* = 0.07) while in‐hospital ICU transfer was lower (6.3% [49/777] to 3.5% [50/1416], aOR: 0.65, 95% CI: 0.44–0.96, *p* = 0.01). In multivariable models to evaluate for predictors of 30‐day readmission and 30‐day readmission combined with 30‐day mortality, younger age and higher CCI score were associated with higher readmission rates and male and higher CCI score were associated with higher combined readmission and mortality, but azithromycin use, shorter duration, and amoxicillin use were not significantly associated with either clinical outcome (Supporting Information S1: Appendix). When combining 30‐day hospital readmission and 30‐day mortality rates, there was no significant difference comparing pre‐ to postintervention (12.9% [100/777)] to 13.8% [195/1416], aOR: 1.12, 95% CI: 0.86–1.45, *p* = 0.40).

## DISCUSSION

Updates to an existing CAP order set were associated with lower total antibiotic duration, lower empiric atypical antibiotic therapy, higher use of narrower spectrum antibiotics, and improved laboratory utilization for non‐ICU hospitalized patients with similar clinical outcomes. Our study adds practical real‐world experience to the growing literature supporting shortened antibiotic durations and avoiding atypical coverage for non‐ICU patients hospitalized with CAP. Additionally, our work demonstrates that stewardship‐guided clinical practices can be sustained and enhanced by continually refining interventions based on new data.

The median antibiotic duration in our study decreased from 6 days to 5 days, with a significantly higher number of patients receiving fewer than 5 days of antibiotics. Currently published CAP guidelines recommend a minimum of 5–7 days of antibiotics, yet several studies show similar clinical efficacy and a trend toward fewer antibiotic‐associated adverse events with even shorter durations.^9^, ^10^ For example, two prospective randomized trials evaluating a 3‐day duration versus 8 days showed no difference in clinical success.^9^, ^10^ Despite the order set recommending 3 days of antibiotics for most patients, the majority still received 5 or more days. This may be related to several reasons. First, it may have taken some patients longer to reach clinical stability, and we hypothesize that many physicians feel uncomfortable discontinuing antibiotics before patients' discharge from the hospital or discharging patients without antibiotics. Second, the order set used for patients admitted through the ED remained low (approximately 38% in both cohorts), yet hospitalist teams reviewed antibiotics daily with medicine pharmacists who recommended antibiotic alignment with the order set. This deserves further investigation; however, our data are suggestive of cultural shifts toward reduced antibiotic duration and add to the growing body of literature suggesting shorter antibiotic durations appear to be safe and effective.

Our study adds to the growing evidence that azithromycin is likely not needed for all non‐ICU patients admitted with CAP. The role of empiric atypical anti‐bacterial therapy for non‐ICU patients with CAP is debated,^13^ with recommendations for atypical coverage present in CAP guidelines in the United States but absent in European guidelines.^17^ Three randomized controlled trials evaluating the role of empiric β‐lactam‐macrolide combination therapy versus β‐lactam monotherapy for hospitalized patients with CAP did not find a mortality difference.^9^, ^15^, ^19^ Results are conflicting regarding the time to clinical resolution but suggest faster clinical resolution in those with more severe disease or confirmed infection with *Legionella* spp. Based on these data, the known diagnostic uncertainty around CAP, and data demonstrating potential harms associated with macrolide therapy, our group chose to remove empiric macrolide therapy from our order set. We saw a significant reduction in patients receiving azithromycin in our postintervention period, yet there was no association between 30‐day readmission and azithromycin use on post‐hoc analysis.

Another notable finding in our study was the increased use of narrow‐spectrum antibiotics for CAP. Despite advancements in available diagnostic assays for the detection of potential pathogens, most patients admitted with CAP do not have a pathogen detected.^20^ Amoxicillin is widely recommended as a first‐line agent for CAP in children^21^; yet, US guidelines for adults currently recommend ampicillin + sulbactam, cefotaxime, ceftaroline, or ceftriaxone as empiric therapy for non‐ICU inpatients.^11^ As excess antibiotic use is associated with increased resistance and adverse events, our stewardship team recommended using 1 dose of IV ceftriaxone followed by oral amoxicillin to complete the antibiotic course. This change was supported by local and national data on CAP etiologies, local *Streptococcus pneumonia* susceptibility data (100% penicillin susceptible), and data suggesting that early oral switch for patients with CAP is safe.^22^ We saw a dramatic increase in amoxicillin use without associated clinical harm, supporting early oral switch to narrow antibiotics for non‐ICU patients admitted with CAP.

A small yet important aspect of our order set focused on appropriate laboratory utilization. IDSA guidelines currently recommend against routine blood culture and urine antigen testing for non‐ICU patients as clinical results rarely impact treatment.^11^ Blood culture orders, which had previously been present yet unselected, were removed completely from the order set and *Streptococcus pneumoniae* urine antigen was changed from preselected to unselected. These changes applied the behavioral economic principle of nudging to help sustain stewardship recommendations and were associated with significantly lower test use in the postimplementation period. In both intervention periods, *Legionella* urine testing was present as a preselected order given our recommendation to minimize azithromycin use. We did see a small yet significant decrease in *Legionella* testing in the postintervention period and hypothesize this could be related to a low clinical suspicion for atypical infection.

Although we noticed an increase in both 30‐day ED visits and 30‐day readmissions in the postintervention period, further evaluation on multivariable analysis examining independent risk factors for readmission found no association between any antibiotic use pattern and readmission or mortality (Table 2). Furthermore, 30‐day combined readmission and mortality (which are competing risks) did not change. Finally, despite shorter total antibiotic durations, we saw no change in LOS.

Our study has several limitations. First, this was a retrospective pre‐post study and despite our attempts to evaluate for predictors of clinical outcomes in adjusted models, residual confounding is likely. Second, the order set went live during the COVID‐19 pandemic. Accordingly, the preintervention period included the COVID‐19 pandemic and CAP treatment may have been influenced by management decisions made during that time. We excluded active encounters for COVID‐19 based on the ICD‐10 code, as there were multiple antibiotic recommendations that changed during that time, and patients with COVID‐19 were not felt to have the same physiology as CAP patients. Third, this study was done in a single academic center with a robust antimicrobial stewardship presence and implementing similar protocols elsewhere may be challenging. Fourth, our duration of antibiotic therapy is a combination of calendar days of inpatient antibiotics in addition to days supplied on discharge prescriptions. We have worked with this type of antibiotic data for some time and went through two rounds of data validation for a random subset of patients; however, it is possible some antibiotic prescriptions were not captured correctly, affecting our estimates of duration of therapy. Fifth, our order set usage may be undercounted as we could only track patients who had the order set implemented in the ED, despite it also being embedded in larger order sets. Sixth, in the postintervention period, there was a notable increase in patients with a comorbidity of COPD. It is unclear if this increase represented a difference in coding behavior or the true prevalence of disease; however, no association between COPD comorbidity and readmission or mortality was found on multivariate analysis. Finally, although a multivariate analysis evaluating specific risk factors for readmission and mortality was performed, additional confounders may exist contributing to the higher 30‐day ED and 30‐day readmission rates. In conclusion, we describe the second iteration of a hospitalist and antimicrobial stewardship‐led intervention aimed at improving care for non‐ICU patients with CAP based on evaluation of emerging literature. We found improvement in all antibiotic and laboratory use measures targeted in our order set, without compromising clinical outcomes. Our data suggest antibiotic choice, route, and duration can be safely aligned with stewardship principles in a subset of non‐ICU CAP patients, and we highlight how antimicrobial stewardship interventions can be sustained and improved over time by continually refining interventions and incorporating new evidence.

## CONFLICT OF INTEREST STATEMENT

The authors declare no conflicts of interest.

## Supporting information

CAP Supplement.JHM Revision.
