# Supplementary material for: Streamlining antibiotic use in community acquired pneumonia: A quality improvement initiative
Source: J Hosp Med. 2026 Feb 10;21(8):890–7. doi: 10.1002/jhm.70283 (PMC13432604; doi:10.1002/jhm.70283)
Supplement: Supplementary file 1 — CAP Supplement.JHM Revision. [file JHM-21-890-s001.docx]

Streamlining Antibiotic use in Community Acquired Pneumonia, A Quality Improvement Initiative

**Appendix:**

1. SQUIRE Check list
2. ICD-10 Codes used for inclusion and exclusion criteria
3. Figure 3. Order Set Changes from 2017 to 2021with Expected Outcomes and Actual Outcomes
4. Table 4. Multivariable model for Outcomes of 30-Day Readmission and Combined 30 Day Mortality and Readmission

Revised Standards for Quality Improvement Reporting Excellence (SQUIRE 2.0) September 15, 2015

| **Text Section and Item**  **Name** | **Section or Item Description** |  |
| --- | --- | --- |
| **Title and Abstract** |  |  |
| **1. Title** | Indicate that the manuscript concerns an initiative to improve healthcare (broadly defined to include the quality, safety, effectiveness, patient-centeredness, timeliness, cost,  efficiency, and equity of healthcare) | Page 1 |
| **2. Abstract** | 1. Provide adequate information to aid in searching and indexing 2. Summarize all key information from various sections of the text using the abstract format of the intended publication or a structured summary such as: background, local problem, methods, interventions, results, conclusions | Page 2 |

| **Introduction** | *Why did you start?* |  |
| --- | --- | --- |
| **3. Problem Description** | Nature and significance of the local problem | Page 3 |
| **4. Available knowledge** | Summary of what is currently known about the problem, including relevant previous studies | Page 3 |
| **5. Rationale** | Informal or formal frameworks, models, concepts, and/or theories used to explain the problem, any reasons or  assumptions that were used to develop the intervention(s), and reasons why the intervention(s) was expected to work | Page 3 |
| **6. Specific aims** | Purpose of the project and of this report | Page 3 |
| **Methods** | *What did you do?* |  |
| **7. Context** | Contextual elements considered important at the outset of introducing the intervention(s) | Page 4 |
| **8. Intervention(s)** | 1. Description of the intervention(s) in sufficient detail that others could reproduce it 2. Specifics of the team involved in the work | Page 5 |
| **9. Study of the Intervention(s)** | 1. Approach chosen for assessing the impact of the intervention(s) 2. Approach used to establish whether the observed outcomes were due to the intervention(s) | Page 6 |
| **10. Measures** | 1. Measures chosen for studying processes and outcomes of the intervention(s), including rationale for choosing them, their operational definitions, and their validity and reliability 2. Description of the approach to the ongoing assessment of contextual elements that contributed to the success, failure, efficiency, and cost 3. Methods employed for assessing completeness and accuracy of data | Page 6 |
| **11. Analysis** | 1. Qualitative and quantitative methods used to draw inferences from the data 2. Methods for understanding variation within the data, including the effects of time as a variable | Page 6 |
| **12. Ethical**  **Considerations** | Ethical aspects of implementing and studying the intervention(s) and how they were addressed, including, but not limited to, formal ethics review and potential conflict(s)  of interest | NA |

| **Results** | *What did you find?* |  |
| --- | --- | --- |
| **13. Results** | 1. Initial steps of the intervention(s) and their evolution over time (*e.g.*, time-line diagram, flow chart, or table), including modifications made to the intervention during the project 2. Details of the process measures and outcome 3. Contextual elements that interacted with the intervention(s) 4. Observed associations between outcomes, interventions, and relevant contextual elements 5. Unintended consequences such as unexpected benefits, problems, failures, or costs associated with the intervention(s). 6. Details about missing data | Page 7-8 |
| **Discussion** | *What does it mean?* |  |
| **14. Summary** | 1. Key findings, including relevance to the rationale and specific aims 2. Particular strengths of the project | Page 8 |
| **15. Interpretation** | 1. Nature of the association between the intervention(s) and the outcomes 2. Comparison of results with findings from other publications 3. Impact of the project on people and systems 4. Reasons for any differences between observed and anticipated outcomes, including the influence of context 5. Costs and strategic trade-offs, including opportunity costs | Page 8-10 |
| **16. Limitations** | 1. Limits to the generalizability of the work 2. Factors that might have limited internal validity such as confounding, bias, or imprecision in the design, methods, measurement, or analysis 3. Efforts made to minimize and adjust for limitations | Page 11 |
| **17. Conclusions** | 1. Usefulness of the work 2. Sustainability 3. Potential for spread to other contexts 4. Implications for practice and for further study in the field 5. Suggested next steps | Page 11 |
| **Other information** |  |  |
| **18. Funding** | Sources of funding that supported this work. Role, if any, of the funding organization in the design, implementation,  interpretation, and reporting | NA |

Inclusion ICD-9 & 10 Codes by Epic Grouper

| 481 | Pneumococcal pneumonia (streptococcus pneumoniae pneumonia) (HCC) |
| --- | --- |
| 482 | Pneumonia due to Klebsiella pneumoniae (HCC) |
| 482.1 | Pneumonia due to Pseudomonas (HCC) |
| 482.2 | Pneumonia due to Hemophilus influenzae (H. influenzae) (HCC) |
| 482.3 | Pneumonia due to unspecified Streptococcus (HCC) |
| 482.31 | Pneumonia due to streptococcus, group A (HCC) |
| 482.32 | Pneumonia due to Streptococcus, group b (HCC) |
| 482.39 | Pneumonia due to other streptococcus (HCC) |
| 482.4 | Pneumonia due to Staphylococcus, unspecified (HCC) |
| 482.41 | Methicillin susceptible pneumonia due to Staphylococcus aureus (HCC) |
| 482.42 | Methicillin resistant pneumonia due to Staphylococcus aureus (HCC) |
| 482.49 | Other staphylococcus pneumonia (HCC) |
| 482.81 | Pneumonia due to anaerobes (HCC) |
| 482.82 | Pneumonia due to escherichia coli (E. coli) (HCC) |
| 482.83 | Pneumonia due to other gram-negative bacteria (HCC) |
| 482.84 | Legionnaires' disease (HCC) |
| 482.89 | Pneumonia due to other specified bacteria(482.89) (HCC) |
| 482.9 | Bacterial pneumonia, unspecified |
| 483 | Pneumonia due to Mycoplasma pneumoniae |
| 483.1 | Pneumonia due to Chlamydia |
| 483.8 | Pneumonia due to other specified organism(483.8) |
| 485 | Bronchopneumonia, organism unspecified |
| 486 | Pneumonia, organism unspecified(486) |
| 487 | Influenza with pneumonia |
| A48.1 | Legionnaires' disease (HCC) |
| J11.0 | Influenza due to unidentified influenza virus with unspecified type of pneumonia |
| J13 | Pneumonia due to Streptococcus pneumoniae (HCC) |
| J14 | Pneumonia due to hemophilus influenzae (HCC) |
| J15.0 | Pneumonia due to Klebsiella pneumoniae (HCC) |
| J15.1 | Pneumonia due to Pseudomonas (HCC) |
| J15.20 | Pneumonia due to Staphylococcus, unspecified (HCC) |
| J15.211 | Pneumonia due to methicillin susceptible Staphylococcus aureus (HCC) |
| J15.212 | Pneumonia due to methicillin resistant Staphylococcus aureus (HCC) |
| J15.29 | Pneumonia due to other staphylococcus (HCC) |
| J15.3 | Pneumonia due to Streptococcus, group b (HCC) |
| J15.4 | Pneumonia due to other streptococci (HCC) |
| J15.5 | Pneumonia due to Escherichia coli (HCC) |
| J15.6 | Pneumonia due to other gram-negative bacteria (HCC) |
| J15.7 | Pneumonia due to Mycoplasma pneumoniae |
| J15.8 | Pneumonia due to other specified bacteria (HCC) |
| J15.9 | Unspecified bacterial pneumonia |
| J16.0 | Chlamydial pneumonia |
| J16.8 | Pneumonia due to other specified infectious organisms |
| J18.0 | Bronchopneumonia, unspecified organism |
| J18.8 | Other pneumonia, unspecified organism |

| Exclusion ICD-10 codes | |
| --- | --- |
| E84.0 | Bronchopneumonia associated with cystic fibrosis (HCC) |
| E84.0 | Cystic fibrosis of the lung (HCC) |
| E84.0 | Cystic fibrosis related bronchopneumonia (HCC) |
| E84.0 | Cystic fibrosis with pulmonary exacerbation (HCC) |
| E84.0 | Cystic fibrosis with pulmonary manifestations (HCC) |
| E84.0 | Infective pulmonary exacerbation of cystic fibrosis (HCC) |
| E84.0 | Mucoviscidosis of lung (HCC) |
| E84.0 | Pulmonary cystic fibrosis (HCC) |
| E84.11 | Cystic fibrosis with meconium ileus (HCC) |
| E84.11 | Meconium ileus in cystic fibrosis (HCC) |
| E84.11 | Meconium obstruction of intestine in mucoviscidosis (HCC) |
| E84.11 | Perforation of intestine due to cystic fibrosis with meconium ileus (HCC) |
| E84.19 | Cystic fibrosis with gastrointestinal manifestations (HCC) |
| E84.19 | Cystic fibrosis with intestinal manifestation (HCC) |
| E84.19 | Cystic fibrosis with other intestinal manifestations (HCC) |
| E84.19 | Digestive system manifestation of cystic fibrosis (HCC) |
| E84.19 | Distal intestinal obstruction syndrome due to cystic fibrosis (HCC) |
| E84.19 | Gastrointestinal cystic fibrosis (HCC) |
| E84.19 | Perforation of intestine due to cystic fibrosis with meconium ileus (HCC) |
| E84.8 | Arthralgia associated with cystic fibrosis (HCC) |
| E84.8 | Arthropathy associated with cystic fibrosis (HCC) |
| E84.8 | Arthropathy related to cystic fibrosis (HCC) |
| E84.8 | Cirrhosis due to cystic fibrosis (HCC) |
| E84.8 | Cirrhosis of liver due to classic cystic fibrosis (HCC) |
| E84.8 | Cystic fibrosis with liver disease (HCC) |
| E84.8 | Cystic fibrosis with other manifestations (HCC) |
| E84.8 | Cystic fibrosis-related arthropathy (HCC) |
| E84.8 | Diabetes mellitus related to CF (cystic fibrosis) (HCC) |
| E84.8 | Diabetes mellitus related to cystic fibrosis (HCC) |
| E84.8 | Elevated liver enzymes level due to cystic fibrosis (HCC) |
| E84.8 | Exocrine pancreatic manifestation of cystic fibrosis (HCC) |
| E84.8 | Female infertility due to cystic fibrosis (HCC) |
| E84.8 | Liver disease due to cystic fibrosis (HCC) |
| E84.8 | Male infertility due to cystic fibrosis (HCC) |
| E84.8 | Osteoporosis due to cystic fibrosis (HCC) |
| E84.8 | Otorhinolaryngological manifestation of cystic fibrosis (HCC) |
| E84.8 | Pancreatic insufficiency due to cystic fibrosis (HCC) |
| E84.8 | Polyneuropathy due to classic cystic fibrosis (HCC) |
| E84.8 | Portal hypertension due to cystic fibrosis (HCC) |
| E84.9 | Atypical cystic fibrosis (HCC) |
| E84.9 | Autosomal recessive neonatal hypertrypsinemia (HCC) |
| E84.9 | CF (cystic fibrosis) (HCC) |
| E84.9 | Classical cystic fibrosis (HCC) |
| E84.9 | Cystic fibrosis (HCC) |
| E84.9 | Cystic fibrosis exacerbation (HCC) |
| E84.9 | Cystic fibrosis not affecting current episode of care (HCC) |
| E84.9 | Cystic fibrosis of pancreas (HCC) |
| E84.9 | Cystic fibrosis without meconium ileus (HCC) |
| E84.9 | Cystic fibrosis without mention of meconium ileus (HCC) |
| E84.9 | Cystic fibrosis, gastritis, and megaloblastic anemia syndrome (HCC) |
| E84.9 | Cystic fibrosis, pancreatic (HCC) |
| E84.9 | Cystic fibrosis, unspecified (HCC) |
| E84.9 | Cystic fibrosis-gastritis-megaloblastic anemia syndrome (HCC) |
| E84.9 | Diabetes mellitus due to cystic fibrosis (HCC) |
| E84.9 | Exacerbation of cystic fibrosis (HCC) |
| E84.9 | Fibrocystic disease of pancreas (HCC) |
| E84.9 | Follicular hamartoma-alopecia-cystic fibrosis syndrome (HCC) |
| E84.9 | Lubani-Al Saleh-Teebi syndrome (HCC) |
| E84.9 | Mucoviscidosis (HCC) |
| E84.9 | Mucoviscidosis of pancreas (HCC) |
| E84.9 | Non-pulmonary, non-gastrointestinal cystic fibrosis (HCC) |
| E84.9 | Pancreas fibrocystic disease (HCC) |
| E84.9 | Pancreatic cystic fibrosis (HCC) |
| E84.9 | Perinatal jaundice due to mucoviscidosis (HCC) |
| E84.9 | Subclinical cystic fibrosis (HCC) |
| V42.0 | Car driver injured in collision with two- or three-wheeled motor vehicle in nontraffic accident |
| V42.1 | Car passenger injured in collision with two- or three-wheeled motor vehicle in nontraffic accident |
| V42.6 | Car passenger injured in collision with two- or three-wheeled motor vehicle in traffic accident |
| V42.7 | Person on outside of car injured in collision with two- or three-wheeled motor vehicle in traffic accident |
| V42.9 | Unspecified car occupant injured in collision with two- or three-wheeled motor vehicle in traffic accident |
| Z94.0 | Abnormal findings of undetermined significance on biopsy of transplanted kidney |
| Z94.0 | Chronic kidney disease after donor nephrectomy |
| Z94.0 | Current pregnancy in first trimester with history of kidney transplantation |
| Z94.0 | Current pregnancy in first trimester with history of renal transplant |
| Z94.0 | Current pregnancy in second trimester with history of kidney transplantation |
| Z94.0 | Current pregnancy in second trimester with history of renal transplant |
| Z94.0 | Current pregnancy in third trimester with history of kidney transplantation |
| Z94.0 | Current pregnancy in third trimester with history of renal transplant |
| Z94.0 | Current pregnancy with history of kidney transplantation |
| Z94.0 | Current pregnancy with history of renal transplant |
| Z94.0 | De novo glomerulonephritis following organ transplant |
| Z94.0 | Deceased-donor kidney transplant |
| Z94.0 | Deceased-donor kidney transplant recipient |
| Z94.0 | Diabetes mellitus following renal transplant (HCC) |
| Z94.0 | H/O kidney transplant |
| Z94.0 | H/O living-donor kidney transplantation |
| Z94.0 | H/O maternal renal transplant, currently pregnant |
| Z94.0 | H/O maternal renal transplant, currently pregnant, first trimester |
| Z94.0 | H/O maternal renal transplant, currently pregnant, second trimester |
| Z94.0 | H/O maternal renal transplant, currently pregnant, third trimester |
| Z94.0 | H/O maternal renal transplant, currently pregnant, unspecified trimester |
| Z94.0 | History of kidney transplant |
| Z94.0 | History of living-donor kidney transplantation |
| Z94.0 | History of maternal renal transplant, currently pregnant |
| Z94.0 | History of maternal renal transplant, currently pregnant in first trimester |
| Z94.0 | History of maternal renal transplant, currently pregnant in second trimester |
| Z94.0 | History of maternal renal transplant, currently pregnant in third trimester |
| Z94.0 | History of maternal renal transplant, currently pregnant, first trimester |
| Z94.0 | History of maternal renal transplant, currently pregnant, second trimester |
| Z94.0 | History of maternal renal transplant, currently pregnant, third trimester |
| Z94.0 | History of maternal renal transplant, currently pregnant, unspecified trimester |
| Z94.0 | History of renal transplant |
| Z94.0 | History of renal transplantation |
| Z94.0 | History of simultaneous kidney and pancreas transplant (HCC) |
| Z94.0 | History of transplantation, renal |
| Z94.0 | HTN, kidney transplant related |
| Z94.0 | Hx of kidney transplant |
| Z94.0 | Hx of living-donor kidney transplantation |
| Z94.0 | Hx of maternal renal transplant, currently pregnant |
| Z94.0 | Hx of maternal renal transplant, currently pregnant, first trimester |
| Z94.0 | Hx of maternal renal transplant, currently pregnant, second trimester |
| Z94.0 | Hx of maternal renal transplant, currently pregnant, third trimester |
| Z94.0 | Hx of maternal renal transplant, currently pregnant, unspecified trimester |
| Z94.0 | Hypertension due to kidney transplant |
| Z94.0 | Immunosuppressive management encounter following kidney transplant |
| Z94.0 | Kidney replaced by transplant |
| Z94.0 | Kidney transplant recipient |
| Z94.0 | Kidney transplant status |
| Z94.0 | Kidney transplant status, cadaveric |
| Z94.0 | Kidney transplant status, living related donor |
| Z94.0 | Kidney transplant status, living unrelated donor |
| Z94.0 | Kidney transplanted |
| Z94.0 | Kidney wrapped in mesh during transplant |
| Z94.0 | Living related donor renal transplant |
| Z94.0 | Living unrelated donor renal transplant |
| Z94.0 | Living-donor kidney transplant recipient |
| Z94.0 | Maternal renal transplant, hx of, currently pregnant |
| Z94.0 | Maternal renal transplant, hx of, currently pregnant, first trimester |
| Z94.0 | Maternal renal transplant, hx of, currently pregnant, second trimester |
| Z94.0 | Maternal renal transplant, hx of, currently pregnant, third trimester |
| Z94.0 | Maternal renal transplant, hx of, currently pregnant, unspecified trimester |
| Z94.0 | Normal renal function of transplanted kidney |
| Z94.0 | Post-transplant osteodystrophy |
| Z94.0 | Presence of transplanted kidney |
| Z94.0 | Reinsertion of Foley catheter following kidney transplantation |
| Z94.0 | Reinsertion of urinary catheter following kidney transplantation |
| Z94.0 | Renal transplant recipient |
| Z94.0 | Renal transplant, status post |
| Z94.0 | S/p cadaver renal transplant |
| Z94.0 | S/P kidney transplant |
| Z94.0 | S/P living-donor kidney transplantation |
| Z94.0 | S/P renal autotransplant |
| Z94.0 | Stage 5 chronic kidney disease with transplanted kidney (HCC) |
| Z94.0 | Status post deceased-donor kidney transplantation |
| Z94.0 | Status post kidney autotransplantation |
| Z94.0 | Status post kidney transplant |
| Z94.0 | Status post living-donor kidney transplantation |
| Z94.0 | Status post renal autotransplantation |
| Z94.0 | Status post simultaneous kidney and pancreas transplant (HCC) |
| Z94.0 | Stenosis of ureter of transplanted kidney not physiolly significant |
| Z94.0 | Stenosis of ureter of transplanted kidney not physiologically significant |
| Z94.0 | Tear of renal vein in previously transplanted kidney |
| Z94.0 | Tear of renal vein in previously transplanted kidney, initial encounter |
| Z94.0 | Tear of renal vein in previously transplanted kidney, sequela |
| Z94.0 | Tear of renal vein in previously transplanted kidney, subsequent encounter |
| Z94.0 | Tear of renal vein in previously transplanted kidney, unspecified laterality, initial encounter |
| Z94.0 | Tear of renal vein in previously transplanted kidney, unspecified laterality, sequela |
| Z94.0 | Tear of renal vein in previously transplanted kidney, unspecified laterality, subsequent encounter |
| Z94.0 | Thrombotic microangiopathy presumed due to infarcted transplanted kidney (HCC) |
| Z94.0 | Transplanted kidney |
| Z94.0 | Ureteral stenosis of kidney transplant, not physiologically significant |
| Z94.1 | H/O heart transplant (HCC) |
| Z94.1 | Heart recipient (HCC) |
| Z94.1 | Heart replaced by transplant (HCC) |
| Z94.1 | Heart transplant recipient (HCC) |
| Z94.1 | Heart transplant status (HCC) |
| Z94.1 | Heart transplant, heterotopic, status (HCC) |
| Z94.1 | Heart transplant, orthotopic, status (HCC) |
| Z94.1 | Heart transplanted (HCC) |
| Z94.1 | History of heart transplant (HCC) |
| Z94.1 | Hx of heart transplant (HCC) |
| Z94.1 | Presence of transplanted heart (HCC) |
| Z94.1 | S/P heterotopic heart transplant (HCC) |
| Z94.1 | S/P orthotopic heart transplant (HCC) |
| Z94.1 | Status post heart transplant (HCC) |
| Z94.1 | Status post heart transplantation (HCC) |
| Z94.1 | Status post heterotopic heart transplant (HCC) |
| Z94.1 | Status post orthotopic heart transplant (HCC) |
| Z94.1 | Status post transplant, heart (HCC) |
| Z94.1 | Transplanted heart (HCC) |
| Z94.2 | H/O lung transplant (HCC) |
| Z94.2 | History of lung transplant (HCC) |
| Z94.2 | History of lung transplantation (HCC) |
| Z94.2 | History of transplantation, lung (HCC) |
| Z94.2 | Hx of lung transplant (HCC) |
| Z94.2 | Lung replaced by transplant (HCC) |
| Z94.2 | Lung transplant recipient (HCC) |
| Z94.2 | Lung transplant status (HCC) |
| Z94.2 | Lung transplant status, bilateral (HCC) |
| Z94.2 | Lung transplant status, lobar cadaveric (HCC) |
| Z94.2 | Lung transplant status, lobar living donor (HCC) |
| Z94.2 | Lung transplanted (HCC) |
| Z94.2 | Presence of transplanted lung (HCC) |
| Z94.2 | S/P lung transplant (HCC) |
| Z94.2 | Status post lung transplantation (HCC) |
| Z94.2 | Status post transplant, lung (HCC) |
| Z94.2 | Transplanted, lung (HCC) |
| Z94.3 | H/O heart and lung transplant (HCC) |
| Z94.3 | Heart and lungs transplant status (HCC) |
| Z94.3 | Heart/lung transplant status (HCC) |
| Z94.3 | Heart-lung transplant recipient (HCC) |
| Z94.3 | History of heart and lung transplant (HCC) |
| Z94.3 | Hx of heart and lung transplant (HCC) |
| Z94.3 | Past history of heart and lung transplant (HCC) |
| Z94.3 | Past hx of heart and lung transplant (HCC) |
| Z94.3 | S/P heart and lung transplant (HCC) |
| Z94.3 | Status post heart and lung transplant (HCC) |
| Z94.3 | Status post transplant, heart and lung (HCC) |
| Z94.4 | Alcohol abuse following liver transplant (HCC) |
| Z94.4 | Biliary anastomotic size mismatch (HCC) |
| Z94.4 | Common bile duct stenosis of transplanted liver (HCC) |
| Z94.4 | Encounter for immunosuppression management after liver transplant (HCC) |
| Z94.4 | H/O liver transplant (HCC) |
| Z94.4 | History of liver recipient (HCC) |
| Z94.4 | History of liver transplant (HCC) |
| Z94.4 | History of liver transplant with complex vascular reconstruction (HCC) |
| Z94.4 | History of transplantation, liver (HCC) |
| Z94.4 | Hx of liver transplant (HCC) |
| Z94.4 | Immunosuppressive management encounter following liver transplant (HCC) |
| Z94.4 | Liver replaced by transplant (HCC) |
| Z94.4 | Liver transplant recipient (HCC) |
| Z94.4 | Liver transplant status (HCC) |
| Z94.4 | Liver transplanted (HCC) |
| Z94.4 | Presence of transplanted liver (HCC) |
| Z94.4 | Recipient of liver transplantation (HCC) |
| Z94.4 | S/P biliary anastomos, choledochocholedochostomy, liver trnsplnt stent (HCC) |
| Z94.4 | S/P biliary anastomosis with size mismatch (HCC) |
| Z94.4 | S/P biliary anastomosis with T-tube placed at time of liver transplant (HCC) |
| Z94.4 | S/P biliary anastomosis without stent or T-tube at time of liver transplant (HCC) |
| Z94.4 | S/P biliary anastomosis wo stent or T-tube at time of liver transplant (HCC) |
| Z94.4 | S/P biliary anastomosis, choledochocholedochostomy, liver transplant stent (HCC) |
| Z94.4 | S/P biliary duct dilatation of transplanted liver (HCC) |
| Z94.4 | S/P biliary duct dilation of transplanted liver (HCC) |
| Z94.4 | S/P choledochocholedochostomy with stent done at time of liver transplant (HCC) |
| Z94.4 | S/P dilation of common bile duct of transplanted liver (HCC) |
| Z94.4 | S/P liver transplant (HCC) |
| Z94.4 | S/P liver transplant with biliary anastomosis with size mismatch (HCC) |
| Z94.4 | S/P liver transplant with biliary anastomosis with T-tube (HCC) |
| Z94.4 | S/P liver transplant, biliary anastomosis without stent or T-tube (HCC) |
| Z94.4 | S/P liver transplant, choledochocholedochostomy with stent (HCC) |
| Z94.4 | S/P liver transplant, Roux-en-Y with stent (HCC) |
| Z94.4 | S/P liver transplant, Roux-en-Y without stent (HCC) |
| Z94.4 | S/P Roux-en-Y with stent done at time of liver transplant (HCC) |
| Z94.4 | S/P Roux-en-Y without stent done at time of liver transplant (HCC) |
| Z94.4 | Status post biliary anastomosis and choledochocholedochostomy with stent done at time of liver transplant (HCC) |
| Z94.4 | Status post biliary anastomosis with size mismatch (HCC) |
| Z94.4 | Status post biliary anastomosis with T-tube placed at time of liver transplant (HCC) |
| Z94.4 | Status post biliary anastomosis without stent or T-tube at time of liver transplant (HCC) |
| Z94.4 | Status post biliary duct dilatation of transplanted liver (HCC) |
| Z94.4 | Status post biliary duct dilation of transplanted liver (HCC) |
| Z94.4 | Status post choledochocholedochostomy with stent done at time of liver transplant (HCC) |
| Z94.4 | Status post dilatation of common bile duct of transplanted liver (HCC) |
| Z94.4 | Status post liver transplant (HCC) |
| Z94.4 | Status post liver transplant with biliary anastomosis without stent or T-tube (HCC) |
| Z94.4 | Status post liver transplant with choledochocholedochostomy with stent (HCC) |
| Z94.4 | Status post liver transplant with Roux-en-Y with stent (HCC) |
| Z94.4 | Status post liver transplant, biliary anastomosis with T-tube (HCC) |
| Z94.4 | Status post liver transplant, biliary anastomotic size mismatch (HCC) |
| Z94.4 | Status post liver transplant, with Roux-en-Y without stent (HCC) |
| Z94.4 | Status post liver transplantation (HCC) |
| Z94.4 | Status post Roux-en-Y with stent done at time of liver transplant (HCC) |
| Z94.4 | Status post Roux-en-Y without stent done at time of liver transplant (HCC) |
| Z94.4 | Stenosis of common bile duct of transplanted liver (HCC) |
| Z94.4 | Stenosis of right hepatic artery of transplanted liver (HCC) |
| Z94.4 | Transplanted liver (HCC) |
| Z94.81 | Abnormal growth post bone marrow transplant, height < expected for age (HCC) |
| Z94.81 | Autologous bone marrow transplantation status (HCC) |
| Z94.81 | Bone marrow replaced by transplant |
| Z94.81 | Bone marrow transplant status (HCC) |
| Z94.81 | H/O allogeneic bone marrow transplant (HCC) |
| Z94.81 | H/O bone marrow transplant (HCC) |
| Z94.81 | History of allogeneic bone marrow transplant (HCC) |
| Z94.81 | History of bone marrow transplant (HCC) |
| Z94.81 | History of bone marrow transplant as a child (HCC) |
| Z94.81 | History of bone marrow transplant within last 6 months (HCC) |
| Z94.81 | History of bone marrow transplant within the past 12 months (HCC) |
| Z94.81 | Hx of allogeneic bone marrow transplant (HCC) |
| Z94.81 | Hx of bone marrow transplant (HCC) |
| Z94.81 | S/P allogeneic bone marrow transplant (HCC) |
| Z94.81 | S/P autologous bone marrow transplantation (HCC) |
| Z94.81 | S/P bone marrow transplant (HCC) |
| Z94.81 | Status post allogeneic bone marrow transplant (HCC) |
| Z94.81 | Status post autologous bone marrow transplant (HCC) |
| Z94.81 | Status post autologous bone marrow transplantation (HCC) |
| Z94.81 | Status post bone marrow transplant (HCC) |
| Z94.81 | Transplanted bone marrow present (HCC) |
| Z94.82 | H/O intestine transplant (HCC) |
| Z94.82 | History of intestine transplant (HCC) |
| Z94.82 | Hx of intestine transplant (HCC) |
| Z94.82 | Intestine transplant status (HCC) |
| Z94.82 | Intestines replaced by transplant (HCC) |
| Z94.82 | Organ or tissue replaced by transplant, intestines (HCC) |
| Z94.82 | S/P intestinal transplant (HCC) |
| Z94.82 | S/P small bowel transplant (HCC) |
| Z94.82 | S/P transplantation of small intestine (HCC) |
| Z94.82 | Status post small bowel transplant (HCC) |
| Z94.83 | Dysuria after pancreas transplant using bladder drainage technique (BDT) (HCC) |
| Z94.83 | Embedded bladder stones after pancreas transplant using bladder drainage technique (BDT) (HCC) |
| Z94.83 | H/O pancreas transplant (HCC) |
| Z94.83 | Haemorrhage after conversion to enteric drainage technique (EDT) for pancreas transplant |
| Z94.83 | Hemorrhage after conversion to enteric drainage technique (EDT) for pancreas transplant (HCC) |
| Z94.83 | History of pancreas transplant (HCC) |
| Z94.83 | History of simultaneous kidney and pancreas transplant (HCC) |
| Z94.83 | Hx of pancreas transplant (HCC) |
| Z94.83 | Pancreas replaced by transplant (HCC) |
| Z94.83 | Pancreas transplant status (HCC) |
| Z94.83 | Pancreas transplanted (HCC) |
| Z94.83 | Received pancreas from donor with hepatitis C (HCC) |
| Z94.83 | S/P pancreatic islet cell transplantation (HCC) |
| Z94.83 | Splenic artery thrombosis after pancreas transplant (HCC) |
| Z94.83 | Status post pancreas transplantation (HCC) |
| Z94.83 | Status post pancreatic islet cell transplantation (HCC) |
| Z94.83 | Status post simultaneous kidney and pancreas transplant (HCC) |
| Z94.83 | Urethritis after pancreas transplant using bladder drainage technique (BDT) (HCC) |
| Z94.89 | Cord blood transplantation status |
| Z94.89 | History of pancreatic islet cell transplantation |
| Z94.89 | History of transplantation of extremity |
| Z94.89 | Other specified organ or tissue replaced by transplant |
| Z94.89 | Other specified organ or tissue replaced by transplant(V42.89) |
| Z94.89 | Other transplanted organ and tissue status |
| Z94.89 | S/P cord blood transplantation |
| Z94.89 | S/P hand transplantation |
| Z94.89 | Status post cord blood transplantation |
| Z94.89 | Status post hand transplantation |
| Z94.89 | Transplant recipient |
| Z94.89 | Transplant recipients |

Table 3. Order Set Changes from 2017 to 2021with Expected Outcomes and Actual Outcomes

|  | **Original** | **Updated** | **Expected outcome** | **Actual outcome** |
| --- | --- | --- | --- | --- |
| **Antibiotics** | Azithromycin x 1 day | No azithromycin | Decreased Azithromycin use | Azithromycin orders decreased from 62.4% (485/777) to 39.4% (558/1416) |
|  | Ceftriaxone x 1 day | No change | No change in empiric [or day 1] ceftriaxone use | Did not evaluate |
|  | Automatic de-escalation to cefuroxime on day 2 | Automatic de-escalation to amoxicillin on day 2 | Increased amoxicillin use | Amoxicillin orders increased from 5.1% (40/777) to 51.1% (723/1416) |
|  | Total antibiotic duration of 5 days | Total antibiotic duration of 3 days | Decreased total duration of antibiotics | Median total antibiotic duration decreased from 6 days (IQR 5,7) to 5 days (4,7) |
| **Labs** | Streptococcus pneumonia urine antigen present and pre-selected | Streptooccus pneumonia urine antigen present and unselected | Decrease in ordering | Streptococcus antigen orders decreased from 72.5% (563/777) to 45.6% (646/1416) |
|  | Procalcitonin present and pre-selected | No change | No change in ordering | No change in procalcitonin orders (69.6% [541/777] to 71.1% [1007/1416]) |
|  | Legionella pneumophilia urine antigen present and pre-selected | No change | No change in ordering | Decreased Legionella urine orders from 72.3% (563/777) to 67.2% (952/1416) |
|  | Blood culture orders present and unselected | Blood culture orders no longer present | Decrease in ordering | Decreased blood culture orders from 62.9% (489/777) to 54% (765/1416) |
|  | Anaerobic Respiratory Culture with Gram Stain present and unselected | No change | No change in ordering | No change in Respiratory culture orders (29.7% [231/777] to 30.9% [437/1416]) |

*For patients admitted to non-ICU units with CAP who do not have a history of antibiotic resistant infection in the past year

Table 4. Multivariable model for Outcomes of 30-Day Readmission and Combined 30 Day Mortality and Readmission

| Outcome | Predictors | Odds ratio (95% CI) | p |
| --- | --- | --- | --- |
|  | Azithromycin | 0.96 (0.70-1.30) | 0.78 |
|  | Amoxicillin as antibiotic | 1.12 (0.80-1.55) | 0.51 |
|  | Antibiotic duration | 1.06 (1.0-1.14) | 0.06 |
|  | Age | 0.98 (0.97-0.99) | <0.01 |
|  | Male | 1.29 (0.94-1.76) | 0.12 |
| 30-Day Readmission | CCI score | 1.11 (1.05-1.17) | <0.01 |
|  | Severity Index | 1.24 (0.88-1.75) | 0.23 |
|  | Heart failure comorbidity | 1.15 (0.65-2.01) | 0.64 |
|  | COPD comorbidity | 1.42 (0.86-2.36) | 0.17 |
|  | Respiratory Virus Season | 0.87 (0.63-1.21) | 0.40 |
|  |  |  |  |
|  | Azithromycin | 0.87 (0.68-1.12) | 0.29 |
|  | Amoxicillin as antibiotic | 0.76 (0.57-1.0) | 0.05 |
|  | Antibiotic duration | 1.02 (0.97-1.08) | 0.43 |
|  | Age | 1 (0.99-1.01) | 0.82 |
|  | Male | 1.31 (1.02-1.69) | 0.04 |
| Combined 30-Day Readmission + Mortality | CCI score | 1.12 (1.07-1.17) | <0.01 |
|  | Severity Index | 1.24 (0.94-1.63) | 0.13 |
|  | Heart failure comorbidity | 0.72 (0.44-1.17) | 0.19 |
|  | COPD comorbidity | 1.04 (0.68-1.61) | 0.85 |
|  | Respiratory Virus Season | 0.86 (0.66-1.13) | 0.28 |

Abbreviations: Chronic Obstructive Pulmonary Disease (COPD), Charlson Comorbidity Index (CCI),
